# Draft Genome Sequence of *Streptomyces* sp. Strain PSAA01, Isolated from the Soil of Eastern Himalayan Foothills

**DOI:** 10.1128/mra.00370-22

**Published:** 2022-06-27

**Authors:** Prasenjit Das, Biraj Sarkar, Amit Ghati, Rittick Mondal, Paulami Dam, Octávio L. Franco, Marlon H. Cardoso, Ashwani Sharma, Shambhu Swarnakar, Florina Miere (Groza), Debnirmalya Gangopadhyay, Sukhendu Mandal, Ahmet Kati, Amit Kumar Mandal

**Affiliations:** a Laboratory of Molecular Bacteriology, Department of Microbiology, University of Calcutta, Kolkata, India; b Department of Microbiology, Barrackpore Rastraguru Surendranath College, Kolkata, India; c Chemical Biology Laboratory, Department of Sericulture, Raiganj University, North Dinajpur, West Bengal, India; d S-Inova Biotech, Programa de Pós-graduação em Biotecnologia, Universidade Católica Dom Bosco, Campo Grande, Brazil; e Centro de Análises Proteômicas e Bioquímicas, Programa de Pós-graduação em Ciências Genômicas e Biotecnologia, Universidade Católica de Brasília, Brasília, Brazil; f Insight Biosolutions, Rennes, France; g Department of Botany, Raiganj University, North Dinajpur, West Bengal, India; h Department of Preclinical Disciplines, Faculty of Medicine and Pharmacy, University of Oradea, Oradea, Romania; i Department of Sericulture, Raiganj University, North Dinajpur, West Bengal, India; j Experimental Medicine Research and Application Center, University of Health Sciences Turkey, Uskudar, Istanbul, Turkey; k Department of Biotechnology, Institution of Health Sciences, University of Health Sciences Turkey, Uskudar, Istanbul, Turkey; University of Arizona

## Abstract

*Streptomyces* strains are powerhouses for a diverse range of secondary metabolites, including antibiotics, anticancer and immunosuppressive agents, and enzymes. Here, we report the genome sequence of *Streptomyces* sp. strain PSAA01, which was isolated from a soil sample taken in Manas National Park, Assam, India, in the eastern Himalayan foothills of India.

## ANNOUNCEMENT

The genus *Streptomyces* belongs to the family *Actinomycetaceae* (1) and presents around 685 species included in the List of Prokaryotic names with Standing in Nomenclature (LPSN) (2). *Streptomyces* species are generally aerobic, filamentous, and spore forming, and they constitute ~90% of the soil actinobacteria (1, 3). The commencement of sporulation is associated with the production of bioactive secondary metabolites (e.g., antibiotics and antifungals) (1, 4, 5). *Streptomyces* species are the sole producers of antibiotics produced by actinobacteria, accounting for 80% of all antibiotic-producing microorganisms (6–11).

Fine alluvium soil samples from Manas National Park, Assam, India (26.6594°N, 91.0011°E), were collected randomly after digging 10 to 15 cm straight down from the soil surface. The soil samples were pretreated for selective isolation of actinomycetes by CaCO_3_ treatment at 30°C for 7 days, which reduces the molds and yeasts in the soil sample, followed by heat treatment at 65°C for 2 h to suppress the growth of all bacteria except actinomycetes (12). Then, 1 g of the soil sample was dissolved in 1 mL 0.9% NaCl solution, serially diluted, spread on starch casein medium (supplemented with filter-sterilized 50 μg mL^−1^ nystatin and cycloheximide) (13), and incubated for 4 days at 30°C. The individual colonies were picked out aseptically and maintained at −70°C in 20% glycerol. A single purified colony of the isolate PSAA01 was inoculated in 5 mL of sterile International *Streptomyces* Project-2 (ISP-2) broth (14) and incubated at 30°C for 3 to 4 days under shaking conditions at 180 rpm. The genomic DNA of PSAA01 was extracted by the standard phenol-chloroform method (15).

Paired-end libraries were prepared and sequenced using the Illumina NovaSeq 6000 platform (Neuberg Diagnostics Pvt. Ltd., Ahmedabad, India), producing 12,765,796 reads with 2 × 161-bp paired-end read length. The DNA library was prepared using the NEBNext Ultra DNA library preparation kit according to the manufacturer's manual. Final DNA libraries were quantified using a Qubit 4.0 fluorometer (product number Q33238; Thermo Fisher Scientific) with a DNA high-sensitivity (HS) assay kit (product number Q32851; Thermo Fisher Scientific). The insert size of the library was checked using a TapeStation 4150 system (Agilent) with highly sensitive D1000 ScreenTapes (product number 5067-5582; Agilent).

Quality assessment of the raw fastq reads of the sample was performed using FastQC v0.11.9 (default parameters) (16). The raw fastq reads were preprocessed using Fastp v0.20.1 (parameters: –-qualified_quality_phred30 –trim_front15 –trim_front25 –length_required50 –correction –trim_poly_g) (17), followed by quality reassessment using FastQC. The processed paired-end reads were mapped to the pre-KMA indexed NCBI 2019 Genome Build database (https://doi.org/10.25910/5cc7cd40fca8e) using KMA (18). The *de novo* assembly was performed using the Unicycler assembler v0.4.8 (https://github.com/rrwick/Unicycler) with default parameters (19). The annotation was carried out via the NCBI Prokaryotic Genome Annotation Pipeline (PGAP) v5.3 with the methods best-placed reference protein set and GeneMarkS-2+ (20). The assembly produced a draft genome sequence encompassing 271 contigs. The *N*_50_ value is 86,830 bp, while the *L*_50_ value is 35. The estimated genome size is 9,224,189 bp, with a G+C content of 71.2% and 99× coverage. A total of 8,088 coding sequences were annotated, including 7 rRNA genes (two 5S, one 16S, and four 23S rRNA genes) and 64 tRNA genes. Further research into the PSAA01 genome will likely facilitate understanding of the molecular basis of bioactive secondary metabolite production for therapeutics.

### Data availability.

This whole-genome shotgun project was deposited in NCBI GenBank (accession number JAKKUU000000000). The version described in this paper is the first version, JAKKUU010000000, and consists of sequences JAKKUU010000001 to JAKKUU010000271. The BioProject and BioSample accession numbers are PRJNA800387 and SAMN25243511, respectively. The raw data are available from the Sequence Read Archive (SRA) under accession number SRR18308362.
